# Interplay Between the Intestinal Microbiota and Acute Graft-Versus-Host Disease: Experimental Evidence and Clinical Significance

**DOI:** 10.3389/fimmu.2021.644982

**Published:** 2021-03-16

**Authors:** Tao Hong, Rui Wang, Xiaoqi Wang, Shijie Yang, Weihao Wang, Qiangguo Gao, Xi Zhang

**Affiliations:** ^1^Medical Center of Hematology, Xinqiao Hospital, Third Military Medical University (Army Medical University), Chongqing, China; ^2^State Key Laboratory of Trauma, Burns and Combined Injury, Third Military Medical University (Army Medical University), Chongqing, China; ^3^Department of Cell Biology, College of Basic Medicine, Third Military Medical University (Army Medical University), Chongqing, China

**Keywords:** hematopoietic stem cell transplantation, acute graft-versus-host disease, intestinal microbiota, diversity, strategies

## Abstract

Allogeneic hematopoietic stem cell transplantation (allo-HSCT) is a potentially curative therapy for many hematological disorders and autoimmune diseases, but acute graft-versus-host disease (aGVHD) has remained a major obstacle that limits allo-HSCT and exhibits a daunting mortality rate. The gastrointestinal system is among the most common sites affected by aGVHD. Experimental advances in the field of intestinal microbiota research enhanced our understanding - not only of the quantity and diversity of intestinal microbiota - but also their association with homeostasis of the immune system and disease pathogenesis, including that of aGVHD. Meanwhile, ever-growing clinical evidence suggest that the intestinal microbiota is dysregulated in patients who develop aGVHD and that the imbalance may affect clinical outcomes, indicating a potential predictive role for microbiota dysregulation in aGVHD severity and prognosis. The current animal and human studies investigating the intestinal microbiota in aGVHD and the understanding of the influence and management of the microbiota in the clinic are reviewed herein. Taken together, monitoring and remodeling the intestinal microecology following allo-HSCT may provide us with promising avenues for diagnosing, preventing or treating aGVHD in the clinic.

## Introduction

Malignancies of the hematopoietic system and therapy-refractory autoimmune diseases are frequently associated with high mortality and hence represent the most common indications to perform allogeneic hematopoietic stem cell transplantation (allo-HSCT) (1, 2). However, graft-versus-host disease (GVHD), in which donor-derived T cells recognize host tissues as foreign, causing inappropriate and aberrant immune attacks, remains one of the major limitations to HSCT. Approximately 40-60% of patients receiving allo-HSCT may suffer from GVHD (3–5) with a mortality rate of 15% to 20% (3, 4, 6–9). Clinically, prophylaxis of acute GVHD (aGVHD) involves immunosuppression of donor cells, but there is no standard approach, and it often varies by institution (3). Treatment protocols can also be challenging because therapeutic options are limited, response rates for corticosteroids are only approximately 50%, and response durations are typically brief (6, 7). In addition, several drugs are reported to be effective in patients not responding to corticosteroids, but most data are unconvincing, and combination therapies tried to date have yielded modest or no benefit over corticosteroids alone (10–12). Because of the small number of results from well-designed, large-scale, clinical studies, there is considerable variability in dealing with aGVHD worldwide, which leads to updated consensus recommendations that still have problems (13).

Much work has been done to research the biological mechanisms participating in the pathogenesis of aGVHD, but the specific nature of these interactions has not been fully elucidated, especially the relationship between aGVHD and the intestinal microbiota. The intestinal microbiota has been proven to be critical for maintaining healthy tissues and stimulating immunity (14), and increasing evidence has revealed that dysbiosis in intestinal microbial populations is linked to human disease and defects in immunity (15–18). Recent studies have notably widened our understanding of the interactions between the loss of intestinal bacterial diversity and aGVHD following allo-HSCT. This review provides an update of current knowledge on the cross-talk between them, with the purpose of determining improved prophylactics and therapies for aGVHD based on the role of the intestinal microbiota.

## The Intestinal Tract and Intestinal Microbiota

**Figure 1:**
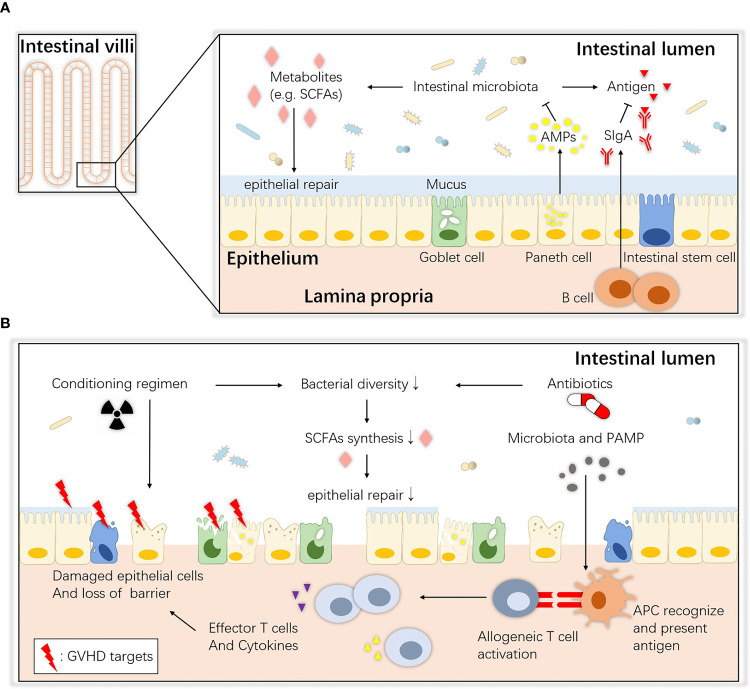
Overview of intestinal ecology and gastrointestinal aGVHD. **(A)** Homeostasis between the commensals and host intestinal epithelium. At steady state, host intestinal epithelial cells live with commensals, and their interaction maintains immune and biological homeostasis. ISCs maintain the regeneration of the epithelium, Paneth cells secrete AMPs that create a sterility gradient, goblet cells produce mucus to separate the microbiota from host epithelial tissue, and immune cells such as B lymphocytes secrete SIgA to neutralize biologically active microbial antigens. Together, they maintain an intact barrier on the mucosa surface. SCFAs (e.g., butyrate) are bacterial fermentation products that can be used as an energy source and regulate the differentiation, recruitment and activation of immune cells. **(B)** Pathogenesis of gastrointestinal aGVHD. During allo-HSCT, the antibiotics and altered diet and cell damage caused by the conditioning regimen all lead to dysbiosis and metabolic disorders. Then, the depletion of SCFAs may also contribute to epithelial defects, allowing translocation of pathogenic bacteria and PAMPs. APCs (e.g., DCs) recognize them and elicit Th1 and Th17 responses and the release of proinflammatory factors that enhance tissue damage. ISCs, intestinal stem cells; AMPs, antimicrobial peptides; SCFAs, short-chain fatty acids; SIgA, secretory immunoglobulin A; APC, antigen-presenting cell; PAMP, pathogen-associated molecular pattern.

The gastrointestinal tract consists of the mucous layer, submucous layer, muscular layer and serosa from the inner to outer layers. The intestinal mucosa, the innermost layer of the gastrointestinal tract, can be further divided into the epithelium, lamina propria and muscularis mucosae (19). The epithelium is a single-cell layer that contains unique secretory cells and stem cells, and many immune cells in the lamina propria help to monitor pathogens and maintain immune tolerance to food and commensal antigens (20). The mucosal surface maintains an intact biological barrier that prevents substantial bacterial and other detrimental invasion into the host tissue and blood circulation under steady-state homeostasis; this function is implemented by epithelial tissues, gut-associated lymphoid tissue (GALT) and important secretory components (21). The commensal intestinal microbiota also contributes to the maintenance of intestinal ecological balance. An overview of homeostasis between the microbiota and the host intestinal mucosa is shown in **Figure 1A**.

The human intestinal tract hosts 10^13^ to 10^14^ microbial organisms of approximately 1000 species (**Table 1**) **(**22, 23). Although viruses and fungi are also present in considerable amounts and diversity, the vast majority of these organisms are bacteria collectively termed the gut microbiota (24–26), which play an important role in the synthesis of a variety of vitamins and amino acids, participating in the metabolism of carbohydrates and proteins and promoting the absorption of various mineral elements (27). The balance and diversity of the gut microbiota is of great importance for the human body, as researchers have found close connections between changes in the gut microbiota and human diseases, such as obesity (28), diabetes (29, 30), functional bowel syndrome (31), autism (32), and autoimmune diseases (e.g., rheumatoid arthritis (33)). For decades, the analysis of the intestinal microbiota has been largely dependent on ex vivo cultivation of bacteria, which yields only 10–30% of the population, limiting knowledge of the bacterial composition (34). In recent years, the development of next-generation sequencing technologies, such as 16S rDNA sequencing and metagenomics, has allowed for further identification of microorganisms, which clarifies the detailed and specific role of the intestinal microbiota in aGVHD, leading to a new era of research (35, 36).

**Table 1:** Bacterial taxonomy of some important microbiota constituents in the literature.

Phylum	Class	Order	Family	Genus	Species
**Verrucomicrobia**	Verrucomicrobiae	Verrucomicrobiales		Akkermansia	
**Proteobacteria**	γ-proteobacteria	Enterobacteriales	Enterobacteriaceae	Escherichia	*E. coli*
**Bacteroidetes**	Bacteroidia	Bacteroidales	Bacteroidaceae	Bacteroide	
**Firmicutes**	Clostridia	Clostridiales	Lachnospiraceae	Blautia	
				Eubacterium	
			Ruminococcaceae		
	Erysipelotrichia	Erysipelotrichiales		Erysipelatoclostridium	
	Bacilli	Lactobacillales	Lactobacteriaceae	Enterococcus	Enterococci
				Lactobacillus	

## Intestinal Microbiota in the Mechanism of aGVHD

### Pathophysiology

Development of aGVHD is considered a three-step process. The microbiota-linked pathogenesis of gastrointestinal aGVHD is summarized herein.

In the first step, when the conditioning regimen of allo-HSCT damages the intestinal epithelium, homeostasis between the host and intestinal commensals is disturbed (**Figure 1B**). Total-body irradiation (TBI) induces dose-dependent damage to the gut lining including killing intestinal stem cells (ISCs), depleting or inhibiting non-epithelial cells, injuring intestinal crypts and causing gastrointestinal tract syndrome (37–39), by mechanisms such as increasing p53-mediated epithelial apoptosis (40) and plasminogen activator inhibitor-type 1 (PAI-1)-mediated enteritis (41). The intestinal mucosa is the major target tissue, and histological evidence has shown villous shortening, increased lymphocytic cell infiltration, crypt destruction and epithelial apoptosis. Crypt cell degeneration has been suggested to be the initial lesion of gastrointestinal aGVHD (42–44), and loss of goblet cells and Paneth cells has been shown to lead to translocation of dominant luminal pathogens and pathogen-associated molecular patterns (PAMPs) as well as intestinal dysbiosis, which further accelerates gastrointestinal aGVHD and infections (45). Moreover, some studies have provided evidence that these toxic effects are partially mediated by the intestinal microbiota. Lai et al. found that mice treated with antibiotics or deficient in myeloid differentiation primary response gene 88 (MyD88), a crucial adaptor for recognition of microbial molecules, showed less crypt loss and less damage to progenitor and stem cells after radiation (46). Seth et al. showed that the microbiota protects against dextran sodium sulfate–induced intestinal damage after radiation (47). It is possible that the microbiota affects the initiation of damage.

**Figure 2:**
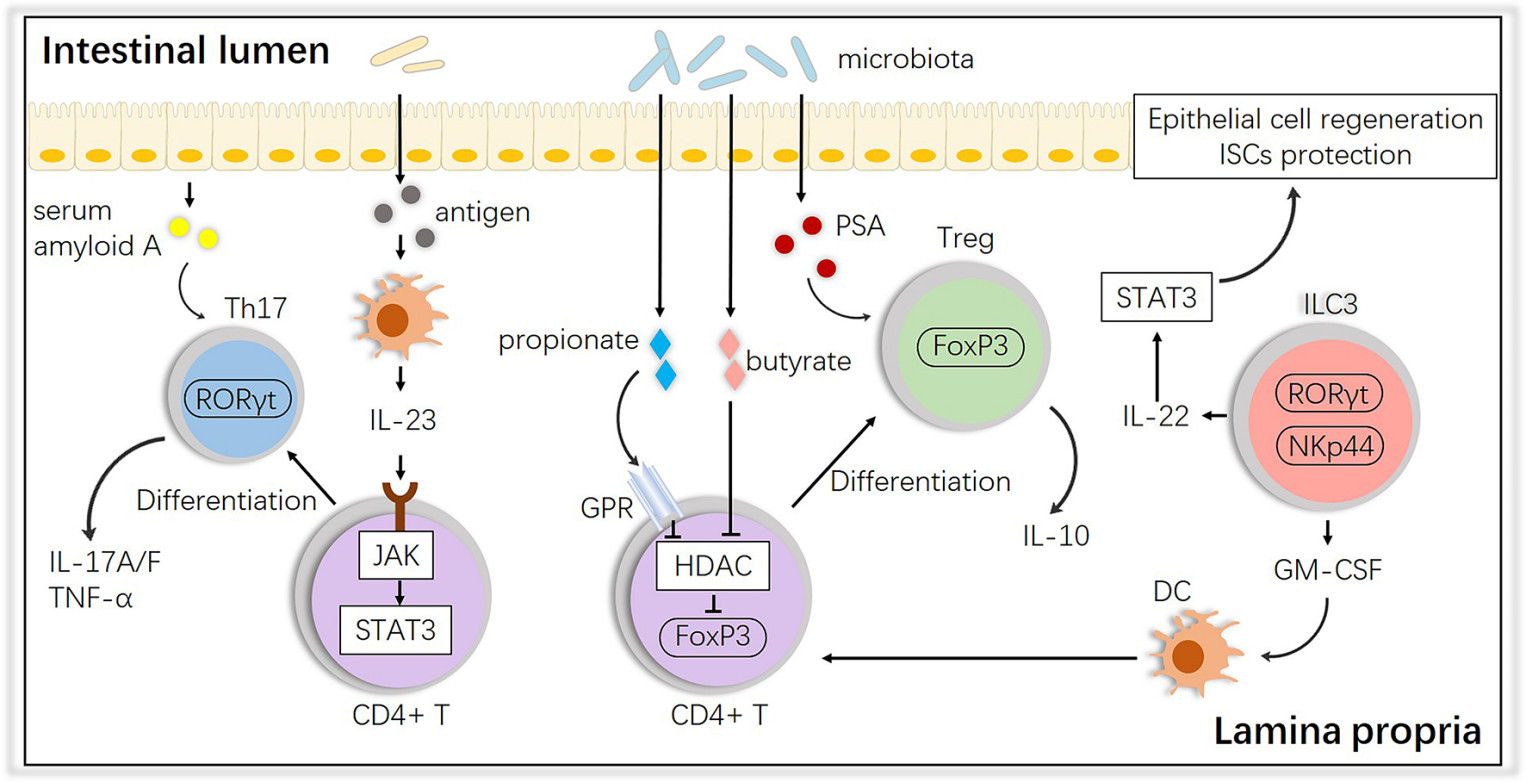
Impact of the intestinal microbiota on T cell subsets and interactions with ILC3s. The intestinal microbiota influences the differentiation of T cells into anti-inflammatory Tregs or proinflammatory Th17 cells, and ILCs play an important role in this process. PSA, polysaccharide A; GM-CSF, granulocyte macrophage colony-stimulating factor; HDAC, histone deacetylase; GPR, G protein receptor.

In terms of the alloreactive cells at the second step, it is well known that potential pathogens and their antigen molecules can activate T cells and affect differentiation. A study showed that cohousing laboratory mice with feral mice produced mice with immune systems closer to those of adult humans, with a preference toward effector and memory T cell populations, suggesting that ‘dirtier’ mice have more intrinsic activation leading to more GVHD (48). After observing their existence, studies have shown that activated T cells access the epithelium *via* gut-specific homing molecules on the T cells and adhesion ligands on the vasculature, such as MAdCAM-1; the intestinal crypts are the primary location invaded by T cells, wherein they directly interact with ISCs (49–54). For T cell differentiation, the induction of both T helper (Th) and regulatory T (Treg) cells is influenced by the microbiota. Breaching the intestinal barrier or penetration by the microbiota in aGVHD activates the IL-23 pathway by JAK-STAT, stimulating epithelial cells to produce serum amyloid A proteins, which leads to Th17 differentiation, an important subset in aggravating aGVHD (55–57). Interestingly, some species, such as *Bacteroides fragilis*, prevent IL-17 production by releasing polysaccharide A (PSA) and promoting Foxp3^+^ Tregs, and Tregs maintain gastrointestinal homeostasis *via* the release of IL-10 (58), which could alleviate aGVHD. Other species, i.e., Clostridiales, produce SCFAs (e.g., butyrate and propionate), which block histone deacetylases (HDACs) through the G-protein receptor (GPR) to promote acetylation of histone H3 in Tregs at the Foxp3 locus, which also induces Treg differentiation (59). In addition, innate lymphoid cells 3 (ILC3s), an activated population with expression of the natural cytotoxicity receptor (NCR, such as NKp44) and nuclear hormone receptor RORγt were found mainly in mucosal tissues and emerged as modulators of conditioning-induced tissue damage in the context of aGVHD, secreting IL-22 and IL-17, which are necessary in defense against bacterial pathogens (60, 61). NCR^+^ ILC3-derived IL-22 has already been found to be crucial in epithelial recovery and protect ISCs from damage by activating STAT3 and downstream regulators of cellular proliferation and survival which finally attenuates aGVHD (62, 63). But there is no direct evidence that NCR^-^ ILC3s-derived IL-17 is involved in the pathology of aGVHD (64). Granulocyte macrophage colony-stimulating factor (GM-CSF) produced by ILC3s is also essential for the normal development of intestinal dendritic cells (DCs) involved in Treg induction (65). These interactions are summarized in **Figure 2**.

The third step links cytokine storms and inflammatory amplification, which induce direct damage and establish typical aGVHD injury. Damage to the intestine plays a central role in amplifying systemic GVHD by propagating a proinflammatory cytokine milieu (66). Moreover, the many combinations of cytokines (e.g., TNF, INF-γ, IL-1, IL-2, and IL-17) and costimulatory networks at the T cell surface are definitely complex, in addition to numerous products produced by the intestinal microbiota. The role of the intestinal microbiota in regulating cytokines has been elucidated in some previous studies. Atarashi et al. showed that on the basis of high potency in enhancing Treg abundance and inducing anti-inflammatory molecules, 17 rationally selected strains of Clostridia result the increase of IL-10 in the gut (67). Another study reported that increased abundance of Enterobacteriaceae is positively correlated with IL-17A aggravating aGVHD (68).

In addition to the abovementioned mechanisms, the pathogenesis of aGVHD involves many other specific mechanisms that require deeper investigation. However, it is increasingly clear that the intestinal microbiota indeed participates in the initiation and development of aGVHD.

### Metabolites

The intestinal microbiota generates a wide range of bioactive metabolites serving as mediators and has pervasive consequences in aGVHD; modifications in bacteria-derived metabolites may be a new perspective regarding this disease (69).

Short-chain fatty acids (SCFAs) are one of the major microbial-derived metabolites found exclusively in the intestinal tract, which function in maintaining the epithelial barrier and colonocyte survival as well as play a diverse array of immune regulatory roles (70, 71). It has been reported that butyrate, one of the three main SCFAs, has a protective effect against aGVHD in murine models; butyrate restoration improved histone acetylation and IEC junctional integrity and decreased IEC apoptosis, ultimately mitigating aGVHD (72–74).

3-Indoxyl sulfate (3-IS), another promising metabolite analyzed in aGVHD, is a tryptophan metabolite of commensal colonic bacteria that has been identified as an indirect marker of a balanced microbiota and predicts the outcome of allo-HSCT (75, 76). Moreover, studies have also shown that gut tryptophan-produced indole metabolites reduce GVHD severity *via* type I interferon (IFN I) (77).

Respiratory metabolites may hold potential as surrogate markers for aGVHD (78–80). Various microbiota constituents are known to produce volatile metabolites, and volatile organic compounds (VOCs) generated during pathologic processes have been reported monitored in diseases such as obesity (81), hepatitis (82) and IBD (83). More recently, Hamilton et al. analyzed the VOCs of patients with and without aGVHD and correctly classified 89% (17 of 19) and 90% (9 of 10) of them, respectively, showing that breath analysis is a feasible and promising noninvasive method to detect and potentially monitor aGVHD (84).

Choline, phosphatidylcholine and carnitine-containing dietary ingredients can be metabolized into trimethylamine (TMA) and subsequently converted into trimethylamine N-oxide (TMAO), which could induce vascular inflammation and endothelial dysfunction (85). Dietary habit such as high-choline diet producing high level of TMAO alters distinct quality and quantity of gut microbiota which might affect microbial metabolites and GVHD severity. The latest research further explored TMAO in aGVHD and found that TMAO enhanced the allogenic GVH reaction. In an animal model, the group stimulated with TMAO showed a worse survival rate, higher GVHD scores and more damage to target tissues, which resulted from Th1 and Th17 differentiation (86).

These studies innovatively provide the link between microbiota-derived metabolites and aGVHD, which sheds light on alleviating aGVHD by controlling metabolism.

## Loss of Microbiota Diversity in aGVHD

Views about the role of the microbiota in aGVHD are separated into two roles: a direct role and an indirect role. The former considers that the intestinal microbiota directly promotes tissue injury or inflammation after allo-HSCT, and animals possess normal immunocompetence but abnormal microbiota community compositions, so eliminating this proinflammatory factor by antimicrobial treatment should protect the recipient from aGVHD (87–89). The latter considers the intestinal microbiota to play an indirect role, as animals raised in germ-free conditions have abnormal development with highly aberrant immunity, and this kind of animal does not possess a prerequisite immune ability to develop aGVHD (90–92). Some researchers even support that both direct and indirect effects may occur simultaneously (93). Furthermore, we can identify some common alterations or differences from animal experiments and clinical studies.

### Animal Studies

Earlier transplantation studies on animals in the 1970s focused on manifestations such as better survival and lower mortality with the management of the intestinal microbiota (94, 95). Subsequently, protection from GVHD in germ-free animals was confirmed in the 1980s (87), including xenogeneic transplantation that typically abrogates severe GVHD (88, 89). Antibiotic-mediated gut decontamination in mice (96) and dogs (97) showed that both microbes and aberrant immune development most proximately affect the development of GVHD. However, few animal studies have measured the impact of antibiotic treatment on microbiota composition or linked specific bacterial species to GVHD at this time.

In the early 21st century, advances in technology advanced research on the alterations (**Table 2**). Shono et al. found that piperacillin-tazobactam and imipenem-cilastatin therapy led to distinct patterns of gut microbiota composition in GVHD mice, with an increasing abundance of an Akkermansia strain, a bacterium with mucus-degrading capabilities, which raises the possibility that mucus degradation may contribute to murine GVHD (102). At the phylum level, the proportions of Firmicutes and Bacteroidetes, two of the major enteric commensals, were found to be decreased in GVHD mice, while the abundance of *E. coli* at the genus level was higher, which could be due to the development of systemic infection (100, 101). Furthermore, for Clostridiales and Lactobacillales, at the order level of Firmicutes, diversity analysis showed loss of the former but expansion of the latter, while eliminating Lactobacillales from mice before transplantation aggravated GVHD, and reintroducing the predominant Lactobacillus species showed a significant protective effect (99). Similar to these results, a report showed that the acute phase of GVHD was characterized by a shift toward the Enterobacteriaceae family. Bacteroides and Enterococcus abundances increased during GVHD, whereas Clostridia, Bifidobacteria and Bacillus were less abundant, but in this report, Lactobacillus abundance was decreased in GVHD (98), Moreover, subsequent studies also presented contrary results that the abundances of Lactobacillus and another uncultured bacterium from Firmicutes increased while the abundances of *E. coli* and another uncultured bacterium from Bacteroidetes decreased in allogeneic immunoglobulin yolk (IgY)-treated GVHD animals (103). However, the exact mechanisms of these alterations remain unclear, although several mechanisms have been proposed in earlier publications, including agglutination, opsonization, and toxin neutralization (106, 107).

**Table 2:** Summary of nearly 10 years of experimental studies investigating the association between alterations in the intestinal microbiota and aGVHD.

Year	Mouse model	Relative abundance alteration of microbiota	Relationship with aGVHD	Ref
2010	(Age- and sex-matched) Balb/c→C57B6(TLR^-/-^, MyD88^-/-^, TRIF^-/-^ and WT)	● *Bacteroides*, Enterococcus ↑ ● Clostridia, Bifidobacteria, Bacillus, Lactobacilli↓	GVHD development is accompanied by shift towards proinflammatory bacterial species(enterobacteria, enterococci and *Bacteroides*/Prevotella).	(98)
2012	B10.BR→B6 Balb/c→B10.BR B6→BM12	● Clostridiales↓ ● Lactobacillales↑	Increased microbial chaos early after allo-HSCT is a potential risk factor for subsequent GVHD.	(99)
2012	(F) B6→B6D2F1 (F) C3H.Sw→B6 (F) B6-Ly5.1→B6D2F1	● Firmicutes, Bacteroidetes↓ ● *E. coli*↑	Diversity of the microbial community was significantly reduced in mice with GVHD.	(100)
2015	(F) B6→BALB.B (F) B6/SJL→BALB.B	● *E. coli*↑	Diversity of the microbial community was significantly reduced in mice with GVHD.	(101)
2016	(F) C57BL/6→129S1	● Erysipelotrichia, Enterococcus, Akkermansia↑ ● Clostridiales↓	Aggravated GVHD mortality was associated with imipenem-cilastatin or piperacillin-tazobactam treatment mice which lead to an increase in Akkermansia muciniphila.	(102)
2017	C57BL/6→B6D2F1 B6D2F1→B6D2F1	● *Lactobacillus* and another uncultured bacterium from Firmicutes↑ ● *E. coli* and another uncultured bacterium from Bacteroidetes↓	Improvement of aGVHD in animals treated with immunoglobulin may be mediated by reducing pathogenic bacteria such as *E. coli* and increasing probiotic bacteria such as *Lactobacillus*.	(103)
2017	BALB/c(WT, IL-17A ^-/-^, IL-17RA ^-/-^)→B6(WT, IL-17A ^-/-^, IL-17RA ^-/-^) BALB/c(WT)→B6(WT, IL-17RA/B/C^-/-^)	● Microbiome of WT mice shifted toward that of the IL-17RA/C–deficient mice during cohousing prior to transplant	IL-17–sensitive microbiota controls susceptibility to aGVHD with increased susceptibility to aGVHD transferred to WT mice *via* cohousing with IL-17RA or IL-17RC–deficient mice.	(57)
2019	(M) C57BL/6→BALB/c (M) C57BL/6→129S1/Sv (M) LP/J→C57BL/6	● Enterococcus↑	Enterococcus expands in mouse after allo-HSCT and exacerbates GVHD severity which is dependent on the lactose.	(104)
2019	(F) BALB/c→B6(WT and Nlrp6^-/-^) (F) C3H.sw→B6(WT and Nlrp6^-/-^)	● Verrucomicrobia, Proteobacteria, Bacteroidetes↑ ● Firmicutes↓	Host NLRP6 play a pathogenic role in aggravating GVHD which was independent of indigenous microbiota changes.	(105)

Collectively, it is evident that not all bacteria have the same effect on GVHD, the composition of bacterial species and their function matter. The perturbations in the diversity and function of bacterial species may be caused by animal strains or research environment. However, one special bacterial species seems to be adverse in GVHD. Enterococci, gram-positive, facultative, anaerobic bacteria, occur in low abundance in the healthy host gut, with reports showing that Enterococcus expansion is associated with increased bloodstream infection and mortality in HSCT (108, 109). The data mentioned above (98) also revealed an increase in enterococci in the development of GVHD. In a recent study (104), in line with previous suspicion, enterococci similarly expanded and dominated the microbiota after allo-HSCT in GVHD mice; lactose drove this growth, and a lactose-free diet attenuated enterococcal expansion and T cell–driven inflammation in GVHD. Indeed, fecal domination by specific translocation of pathogenic bacteria, such as Enterococcus, is a significant risk factor for the development of aGVHD and increased overall GVHD-related mortality. Some easily overlooked factors, such as dietary elements, may play important roles in the progression, which should be closely managed.

The development of biogenetics has led to a focus on specific gene sites. For example, host NOD-like receptor family pyrin domain-containing 6 (NLRP6) regulates microbiota-dependent protection in intestinal colitis and tumorigenesis (110–113), but Tomomi et al. found an inverse effect in GVHD, in which host NLRP6 played a pathogenic role in aggravating intestinal damage (105). Interestingly, this influence was independent of indigenous microbiota changes. Toll-like receptors (TLRs), which sense bacterial lipoproteins and LPS and DNA, are suspected to modulate innate immune responses, and a major role of TLR9-mediated sensing of bacterial DNA in the aggravation of GVHD has been reported (114–116). With gene knockout, Markus et al. similarly proved that bacterial innate immune receptor TLR^-/-^ mice showed significantly reduced GVHD mortality, which was further confirmed by less pronounced GVHD scores over time (98).

Furthermore, some groups have demonstrated substantial differences between the gut microbiota of mice purchased from different commercial vendors or repositories (117). There are clear differences in microbiota composition and diversity between sexes in previous studies (118, 119). In addition, Floris et al. showed that BALB/c mice had higher abundance and diversity of immunoglobulin A (IgAs) than C57BL/6 mice which is correlated with increased microbiota diversity (120). And aging alters the gut microbiota in mice, in which aged microbiome leads to an exaggerated systemic inflammatory response and reduced levels of SCFAs in young mice (121). In conclusion, gut microbiota composition can be influenced by housing, gender, host genetic, age, et al, and further studies are needed to determine the impact of different murine models and strains on aGVHD.

### Human Studies

Early in the 1980s, in a clinical report of 130 patients with aplastic anemia undergoing allo-HSCT, gut decontamination and laminar airflow isolation were shown to lower the incidence of aGVHD (122). However, subsequent clinical research of human aGVHD has demonstrated that loss of intestinal microbiota diversity is associated with aGVHD, as microbiota disruption characterized by expansions of potentially pathogenic bacteria and reduction in alpha diversity (a variable that reflects the number of unique bacterial taxa present and their relative frequencies) have been reported. Some advanced clinical studies performed in recent years may further provide us with a better understanding of these alterations (**Table 3**).

**Table 3:** Summary of human studies investigating the association between the microbiota and GVHD in the past 10 years.

Year	Patients	Relative abundance alteration of microbiota	Outcomes	Ref
2012	18 adult patients (8 GVHD vs. 10 non-GVHD)	● In GVHD patients: Lactobacillales↑ Clostridiales↓	GVHD group had decreased stool microbial diversity, microbial chaos early after transplantation is a potential risk factor for subsequent GVHD.	(99)
2014	80 adult patients	● In GVHD patients: Enterococcus, Streptococcus, *Lactobacillus*↑	Increased mortality from GVHD was associated with lower diversity of microbiota at engraftment, which showed a strong predictive effect on mortality.	(123)
2015	115 adult patients	● In GVHD patients: Blautia↓	Increased abundance of commensal bacteria belonging to the Blautia genus is associated with reduced lethal GVHD and improved OS.	(124)
2017	29 pediatric patients	● In GVHD patients: Enterobacteriaceae, Enterococcus↑ ● In non-GVHD patients: anti-inflammatory Clostridia(AIC), *Bacteroidetes*, Bifidobacterium ↑	Exposure to antianaerobic antibiotics clindamycin lead to depletion of Clostridia species which is associated with GVHD in pediatric HSCT patients.	(125)
2017	66 adult patients (52 GVHD vs. 14 non-GVHD)	● In GVHD patients: oral Actinobacteria, oral Firmicutes↑ Lachnospiraceae↓	The stool microbiota at neutrophil recovery post-HSCT is predictive of subsequent development of aGVHD.	(126)
2018	81 adult patients (32 GVHD vs. 49 non-GVHD)	● In GVHD patients: Enterobacteriaceae↑ Lachnospiraceae, Ruminococcaceae↓	Intestinal microbiota might induce aGVHD by influencing the Treg/Th17 balance.	(68)
2019	141 adult patients (83 grade 0-I aGVHD vs. 58 grade II-IV aGVHD)	● In GVHD patients: Proteobacteria, Gammaproteobacteria, Enterobacteriaceae↑ Firmicutes, Clostridia, Lachnospiraceae, Peptostreptococcaceae, Erysipelotrichaceae, Blautia, Lachnoclostridium, Erysipelatoclostridium, Eubacterium↓	GVHD group had lower diversity of microbiota. The AIM score defined as microbiota diversity of 4 bacterials (Lachnospiraceae, Peptostreptococcaceae, Erysipelotrichaceae, Enterobacteriaceae) was positively correlated with aGVHD grade and could be predictive of the development of aGVHD.	(127)
2019	1325 adult male patients	● In GVHD patients: enterococcal↑	Expansion of enterococcal was associated with GVHD and mortality which can be driven by lactose.	(104)
2020	70 patients (35GVHD vs. 35 non-GVHD)	● In GVHD patients: Lachnospiraceae, Blautia, Ruminococcaceae↓	Microbiota alterations were highly specific of GI aGVHD severity with lower bacterial biomass, a-diversity and decreased butyrate.	(128)
2020	1362 adult patients from 4 centers	● In GVHD patients: Enterococcus, Klebsiella, Escherichia, Staphylococcus, Streptococcus↑	Patterns of microbiota disruption during allo-HSCT were similar across transplantation centers and geographic locations which were characterized by loss of diversity and domination by single taxa, lower diversity was associated with higher risks of TRM and death attributable to GVHD.	(129)

OS, overall survival; AIM score, accumulated intestinal microbiota score; TRM, transplant-related mortality.

In 2012, Jenq et al. studied changes in the microbiota of patients undergoing allo-HSCT and found that only the GVHD group had decreased microbial diversity with increased Lactobacillales abundance and loss of Clostridiales, suggesting that shifts in diversity were a result of GVHD rather than allo-HSCT or antibiotic exposure (99). In 2014, Taur Y et al. found that loss of bacterial diversity in stool specimens was associated with increased mortality from GVHD, and survival at 3 years after allo-HSCT was 36%, 60%, and 67% for patients with low, intermediate, and high microbiota diversity, respectively (123). However, patients in this study received T cell-depleted grafts, which could also reduce GVHD. Jenq et al. further investigated 64 allo-HSCT recipients of T cell–replete grafts and found that a higher abundance of Blautia was associated with a reduced risk of GVHD-related mortality and increased overall survival (124). Blautia is a genus that belongs to the class Clostridia. As reported in previous studies, Clostridiales rescue intestinal epithelial cell damage by upregulating Treg cells through the production of the SCFA butyrate (59, 67, 130), and alteration of the indigenous microbiota with 17 rationally selected strains of high butyrate-producing Clostridia led to decreased GVHD (74). Consistent with these findings, in 2017, Simms et al. reported a significant decline in anti-inflammatory Clostridia in pediatric patients with aGVHD (125). Therefore, it can be speculated that some microbial taxa, such as Blautia, are beneficial for the outcomes of HSCT and mitigation of aGVHD, as they behave as drivers in this process, which should be protected and used in a probiotic approach. By contrast, the Enterococcus genus from Lactobacillales contributes to inflammation, whose role in human aGVHD is the same as that in animals, as expansion of Enterococcus association with increased GVHD in humans has been reported (104, 131). Lactobacillus, another genus of Lactobacillales, showed a possible protective effect in human GVHD (99). In 2018, Lijie Han et al. found that GVHD patients showed a higher abundance of Proteobacteria and a lower abundance of Clostridia, which was correlated with the Treg/Th17 ratio and H3 acetylation, indicating an interaction among alterations in the microbiota, allogenic T cell activation and histone acetylation (68). One year later, this team (127) proved again that in aGVHD, the diversity of the microbiota was significantly lower, with decreases in Clostridia, Lachnospiraceae, Blautia, Eubacterium, and Erysipelatoclostridium abundance and increases in Enterobacteriaceae abundance. This finding was consistent with a study in 2017 showing a persistent lack of Lachnospiraceae and Bacteroidaceae species in GVHD patients, whereas Lachnospiraceae was negatively correlated with neutrophil recovery (126). In addition, the specific actors in the intestinal ecosystem involved in the pathologic process of aGVHD have been explored more recently (128). Shown in stool samples, microbiota alterations were highly specific to gastrointestinal aGVHD severity, and a negative correlation was observed with the Lachnospiraceae, especially the Blautia genus, and Ruminococcaceae families. On the other hand, geographic variations matter, while a recent study analyzing 8767 fecal samples from 1362 patients with allo-HSCT at 4 different centers likewise showed a similar association between lower intestinal diversity and higher risks of transplantation-related death and death attributable to GVHD (129).

From these data, we conclude that restoring intestinal microbiota diversity after allo-HSCT is beneficial in the clinic, protective indigenous probiotics should be preserved to balance the alteration of intestinal microbiota community for patients.

## Clinical Interventions and Value of the Intestinal Microbiota in aGVHD

### Diet

Certain diets may contribute to the development of GVHD given that food is one of the most important factors affecting the composition of the intestinal microbiota (132). One example is a choline diet, and a murine model has shown that a high-choline diet enhances the allogenic GVH reaction, which leads to more aGVHD (86). The effect of parenteral or enteral nutrition on the intestinal ecosystem during HSCT has also been evaluated, with preference toward the latter choice, as enteral feeding has been shown to protect against GVHD in several studies (133–135), while parenteral nutrition was associated with poor outcomes and other complications (136, 137). The type of oral nutrition may be another important factor. Recently, elaborate foods known as a neutropenic diet for allo-HSCT patients have been reexamined, and advanced evidence has shown limited benefit and even potentially harm of supplying aGVHD patients with neutropenic diets (138, 139).

### Prebiotics and Probiotics

Intervention in the intestinal microbiota with a nutritional approach including prebiotics and probiotics may be another promising treatment option for aGVHD.

Prebiotics are indigestible compounds, usually indigestible carbohydrates, that bacteria have an advantage in metabolizing, resulting in the production of SCFAs and metabolites with a potential immunomodulatory role (140, 141). Strategies have been studied in the setting of aGVHD to modulate the intestinal microbiota by supplementation with inulin, oligosaccharides, galacto-oligosaccharides, and potato starch, showing beneficial results. In a recent study, Yoshifuji et al. found that intake of resistant starch and GFO (glutamine, fiber, and oligosaccharide) shortened the duration of oral mucositis and diarrhea and reduced the incidence and severity of aGVHD (142). Other clinical trials focused on fructooligosaccharide, potato-based starch, and gluten-free diets are currently being studied for potential benefit (143).

Probiotics are ingestible formulations of live bacteria that can modulate intestinal homeostasis. A probiotic strategy achieved by FMT consists of introducing one strain or selected strains of microorganisms that confer a benefit. An early report showed that a probiotic-rich diet prior to HSCT is associated with earlier neutrophil engraftment and a shorter duration of febrile neutropenia (144). Some probiotics were found to be safe to administer during aGVHD, such as *Lactobacillus plantarum* reported in pediatric patients (145) and *Lactobacillus rhamnosus* GG in murine models (146). However, controversies exist. Recently, there have been some concerns regarding the safety of administering living microorganisms to immunocompromised patients with altered gut permeability, as some clinical cases have demonstrated sepsis (147), bacteremia (148) and meningitis (149) after treatment of pediatric patients.

### Antibiotics

Given that the intestinal microbiota critically affects transplant outcomes, correctly managing the influence of the microbiota in GVHD—antibiotics has been encouraged. The advent of techniques to generate and maintain germ-free rodents since the 1940s made it possible to examine the microbiota in animals (123), and subsequent studies demonstrated the benefits of using antibiotics. Bekkum and Jones et al. found that germ-free mice housed in sterile conditions or mice treated with antibiotics developed less severe aGVHD (94, 95). Vossen et al. showed that in a cohort of 112 pediatric patients, recipients treated with total gastrointestinal decontamination (GID) using high doses of nonabsorbable antibiotics prevented moderate-to-severe aGVHD, suggesting that the translocation of luminal bacteria and their cell wall-derived compounds might be inhibited during total GID (150). Weber et al. analyzed 394 patients receiving allo-HSCT and found that the treatment of rifaximin correlated with lower enterococcal positivity and higher urinary 3-indoxyl sulfate concentrations. Patients on rifaximin showed lower 1-year transplant-related mortality and higher overall survival. Infectious complications with systemic antibiotic use did not abrogate the beneficial effects of rifaximin on intestinal microbiota composition in the early course of allo-HSCT or outcome (151).

However, the use of gut-decontamination prophylactic antibiotics seems to be a doubled-edged sword. A retrospective analysis in 2018 mentioned above (68) proved that β-lactam antibiotic administration was an independent risk factor for aGVHD according to the Cox regression model for multivariate analysis of aGVHD. Although showing no significant adverse effect, in an allo-BMT murine model, treatment of both donor mice with broad-spectrum antibiotics and control SPF donor mice induced GVHD mortality at a similar rate, and reducing and altering the microbial flora in the donors had no effect on their T cell alloreactivity and induction of GVHD after allogeneic BMT (152). In human studies, the concept that gut decontamination prevents aGVHD is controversial given that some subsequent clinical trials have failed to demonstrate consistent benefits (68, 153–155). Prophylactic use of antibiotics is reported to be associated with more severe aGVHD that involves the intestinal tract and liver, impacting 1-y and 2-y overall survival (OS) in patients receiving myeloablative regimens (155). Earlier antibiotic treatment in patients prior to allo-HSCT was further associated with higher transplant-related mortality than no antibiotics (76). A potential role for anaerobic bacteria, in particular Clostridiales, was supported that Blautia abundance was inversely correlated with the risk of developing gastrointestinal GVHD (124), while clindamycin, an anti-anaerobic bacterial agent, increased the risk of GVHD by depleting anti-inflammatory clostridia (125). Reported previously, piperacillin-tazobactam and imipenem-cilastatin increased the incidence, severity, and mortality of GVHD, and piperacillin-tazobactam reduced Bacteroidetes and Lactobacillus abundance (102). Subsequent evidence further supports the adverse role of anti-anaerobic bacterial penicillin derivatives and carbapenems, as both are associated with a higher incidence of GVHD (156). Although seemingly negative, these findings may enlighten us that antibiotics preserving anaerobic bacteria may potentially reduce the risk of developing gut GVHD.

In conclusion, it is obvious that there remain many controversies for GVHD patients to undergo antibiotic therapy, which needs further exploration. Thus, it is of great necessity to identify new strategies to maintain the diversity and richness of the intestinal microbiota. Different attempts have been made in clinical practice involving narrow-spectrum antibiotics and modulating the timing and duration of treatment.

### FMT

Loss of microbiota diversity creates an opportunity to intervene in aGVHD by reestablishing diversity using microbes that modulate inflammation. FMT has been investigated as a potential therapeutic strategy for gut GVHD in both preventive and therapeutic strategies in recent years. The recent insight of FMT considers it the ‘ultimate probiotic’ for GVHD in allo-HSCT because it directly modifies the host’s intestinal microbiome composition to restore eubiosis and gut homeostasis (157). Indeed, according to some previous reports, FMT significantly improves the outcome of GVHD. Kakihana et al. reported that 4 patients received FMT 92 days after HSCT, all patients responded to FMT within several days, with 3 complete responses and 1 partial response, and an increase in peripheral effector regulatory T cells during the response to FMT was also observed (158). Similarly, another study reported that two of three patients achieved a complete response with multiple FMTs, while the other obtained a partial response; the FMT response was correlated with increased microbial diversity and richness (159). More recent reports further illustrate the role of FMT in GVHD, with promising results (160–162). Currently, a number of ongoing clinical trials to investigate the role of FMT in preventing intestinal GVHD following allo-HSCT, summarized in **Table 4**, are being carried out to try to find the best treatment protocol.

**Table 4:** Summary of ongoing microbiota-linked (not)recruiting clinical trials for GVHD.

Number	Trail title	Interventions	Aims	Study design	Phase	Patients	Time
NCT03819803	Fecal microbiota transplantation in aGVHD after ASCT	FMT	To explore the employment of FMT in GI-aGVHD.	Single group assignment Open label	III	15	2017/3/1-2020/12/31
NCT04285424	FMT for steroid resistant gut acute GVHD	FMT	To evaluate safety and efficacy of FMT for the treatment of steroid resistant GVHD of the gut.	Single group assignment Open label	Early I	30	2020/3/1-2022/3/1
NCT03812705	Fecal microbiota transplantation for steroid resistant/dependent acute GI GVHD	FMT	To evaluate the efficacy and safety of fecal microbiome transplantation in patients with steroid resistant/dependent acute gastrointestinal GVHD.	Single group assignment Open label	II	30	2018/12/13-2022/12/31
NCT04269850	Fecal microbiota transplantation with Ruxolitinib and Steroids as an upfront treatment of severe acute intestinal GVHD	FMT; Ruxolitinib; Methylprednisone	To evaluate this combination treatment in the first line with FMT.	Single group assignment Open label	I/II	20	2019/9/1-2023/9/1
NCT03371667	To compare the efficacy of the addition of Methotrexate (MTX) to current standard acute GVHD first-line treatment with corticosteroids	Methotrexate	To compare the efficacy of the addition of MTX to current standard acute GVHD first-line treatment with corticosteroids.	Randomized Multicenter Double blinded Parallel assignment	III	102	2018/8/16-2021/9
NCT03727113	Optimization of antibiotic treatment in hematopoietic stem cell receptors	Antibiotics	To demonstrate that in ASCT receptors a predefined protocol of optimization of the antibacterial treatment will preserve the intestinal microbiota diversity which will correlate with decrease incidence of acute GVHD.	Observational Case-Control Prospective	Unknown	180	2018/1/16-2020/5/31
NCT03727113	Choosing the Best Antibiotic to Protect Friendly Gut Bacteria During the Course of Stem Cell Transplant	Piperacillin-tazobactam	To compare the effects of different antibiotics on the community of friendly bacteria in the gut	Randomized Parallel Assignment Open Label	II	144	2017/2/10-2021/2
(not R) NCT04059757	Fecal microbiota transplantation for the treatment of gastrointestinal acute GVHD	FMT	To see the efficacy and what side effects are seen with FMT as a treatment for GVHD.	Single group assignment Open label	II	17	2020/7/1-2021/12
NCT04280471	Fecal microbiota transplantation for the treatment of severe acute gut Graft-Versus-Host Disease	FMT capsule	To explore side effects of using an investigational procedure (FMT) in treating patients with severe acute gut GVHD.	Single group assignment Open label	I	10	2020/6/30-2022/7/1
NCT04139577	FMT In high-risk acute GVHD after allo-HCT	FMT	To evaluate the effectiveness of Fecal Microbiota Transplant (FMT) treatment in high-risk acute GVHD.	Single group assignment Open label	I	11	2019/11-2023/5/1
NCT03549676	Fecal microbiota Transplantation for Treatment of Refractory Graft Versus Host Disease-a Pilot Study	FMT	To evaluate safety and efficacy of FMT for the treatment of refractory GVHD of the gut.	Single group assignment Open label	I	15	2019/7/1-2020/12/31
NCT03359980	Treatment of steroid refractory gastro-intestinal acute GVHD after allogeneic HSCT With fecal microbiota transfer	FMT	To explore the employment of FMT in GI-aGVHD.	Single group assignment Open label	II	32	2018/8/13-2020/12

FMT, fecal microbiota transplantation; GI-aGVHD, gastrointestinal acute graft-versus-host disease.

Although many reports have shown that FMT is a safe and effective strategy used in different situations to modulate the microbiota and cure aGVHD, it should be noted that patients are usually immunocompromised with altered intestinal permeability, as infectious complications after FMT have been reported in other settings at the same time (163, 164). Moreover, the evidence mentioned above suggests a beneficial role of FMT in GVHD and needs to be confirmed in larger studies. The optimization of all practical aspects of FMT still needs to be addressed in the future.

### Predictive Marker

As reported in the current literature, a reduction in microbiota diversity and alteration of metabolites can predict transplantation outcomes and aGVHD, shedding light on the value of the microbiota. In 2015, Weber et al. detected 3-indoxyl sulfate (3-IS) in urine specimens within the first 28 days after allo-HSCT in 131 patients and found that a low level of 3-IS within the first 10 days was associated with significantly higher transplant-related mortality and worse overall survival 1 year after allo-HSCT. Furthermore, the class Bacilli was proven to be associated with low 3-IS levels (75). In 2017, Golob et al. showed that a gradient of 20 types of bacterial species could predict severe aGVHD by calculating a gradient of the sum of the relative abundance of positively correlated bacteria minus the sum of the relative abundance of negative correlates (126). Similarly, a study in 2019 by Lijie Han et al. showed that microbiota diversity combined with the gradients of 4 bacteria (Peptostreptococcaceae, Erysipelotrichaceae, Enterobacteriaceae, and Lachnospiraceae) can predict the development and severity of aGVHD (127). Geographic variations in the composition of human microbial communities and differences in clinical practices across institutions raise the question of whether relationships between microbiota composition and clinical outcomes after allo-HSCT are generalizable. In 2020, Peled et al. conducted a study with data from four clinical research institutions by comparing risk scores from regularized Cox regression with cross-validation; they showed that not only a diversity metric but also a signature of specific bacterial abundances was informative about the risk of death after allo-HSCT across institutions (129). Similarly, another study analyzing data from stool and blood samples of 150 patients from two centers who underwent allo-HSCT also showed that gut microbiota score (GMS) from a LASSO (least absolute shrinkage and selection operator) model at neutrophil engraftment could predict aGVHD (165).

Thus, studies on the microbiota as a predictive marker for aGVHD are worth further exploration to provide assistance with the currently available tools for predicting the development of aGVHD.

## Conclusion and Prospects

Alteration of the intestinal microbiota is a corollary given that the gut is an aGVHD target organ. Although we could further explore the intestinal microbiota by better dissecting compositional and functional microbiota structures, most of the research has concentrated on characterization, with data analyzed only *via* correlation. In this regard, determining which specific bacterial taxa are the main taxa affecting aGVHD is of great urgency and importance. In addition to descriptive investigations, mechanistic investigations are needed, which will help translate these associations into therapies for aGVHD if the microbiota is causal.

**Figure 3:**
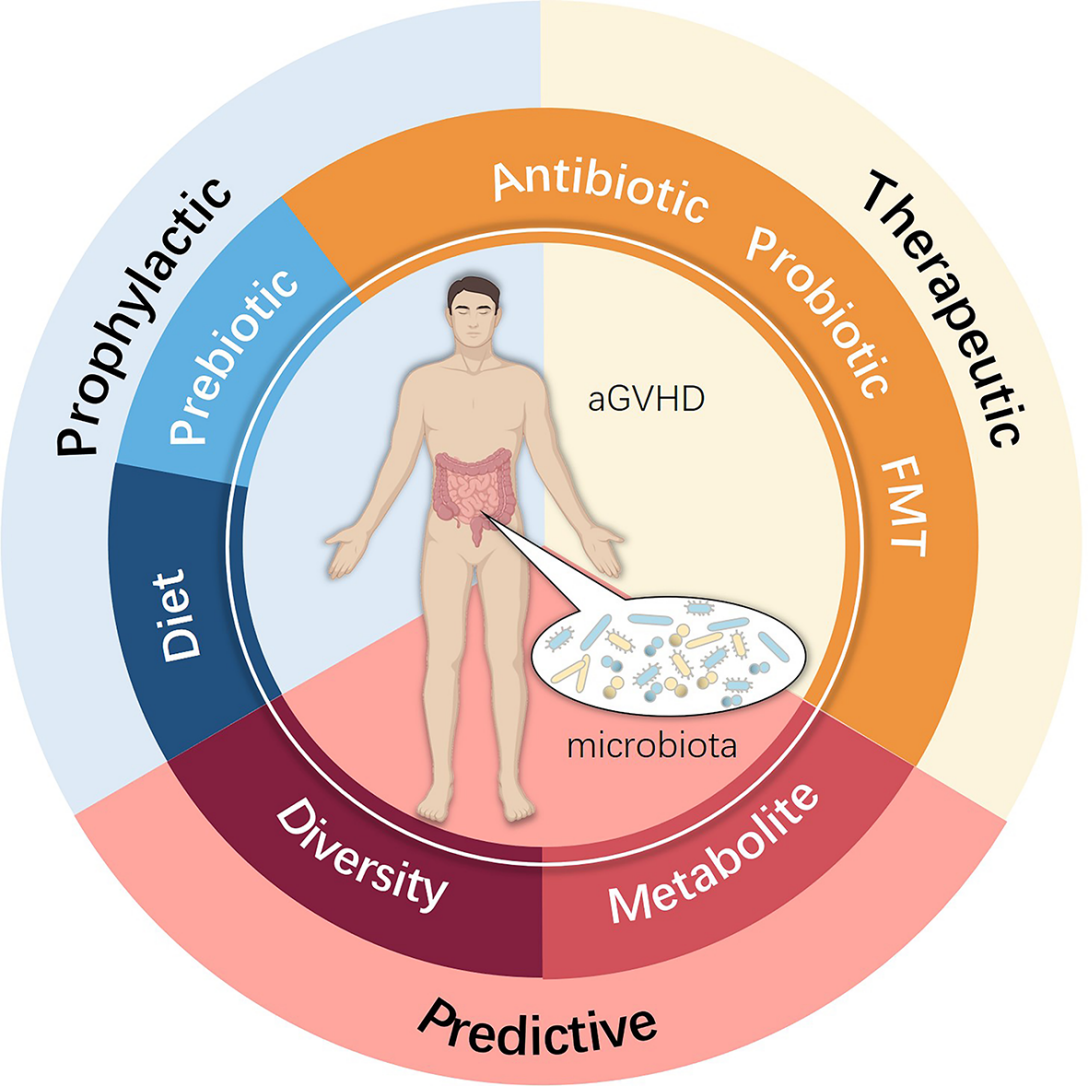
Potential clinical intervention associated with the intestinal microbiota used for preventing, treating and predicting aGVHD.

New approaches to prevent and cure aGVHD remain an unmet need that can be best addressed by understanding the complex pathophysiology, with increasing evidence indicating that the intestinal microbiota indeed participates in this process. Future studies are essential to explore further the role of the intestinal microbiota in aGVHD to elucidate the real impact of microbiota ecology. A promising approach may involve altering certain microbiota species by more precise and safer methods, which may consist of diet, prebiotics, probiotics, advanced antibiotic therapies, FMT, and microbiota metabolism analyses (**Figure 3**). Fully understanding the mechanism by which loss of microbiota diversity influences specific molecules and pathways and regulates the pathogenesis and development of aGVHD remains a top concern. Only by attempting to find better prophylactic and therapeutic schemes for allo-HSCT complications can we focus on what truly matters, which is also the appeal and value of the Human Microbiome Project and precision medicine.

## Author Contributions

The manuscript was conceptualized by XZ and QG. TH wrote the majority of the manuscript. RW and XW co-wrote the manuscript. The figures were designed by TH and drawn by XW and SY. WW, RW, and TH summarized the tables. All authors contributed to the article and approved the submitted version.

## Funding

This work was supported by The 2020 Open Project (Key Project) of the National Center for Clinical Medicine Research on Hematological System Diseases (Grant No. 2020ZKZC02), National Key Research and Development Plan “Stem Cell and Transformation Research” Key Special Project (grant no. 2017YFA0105502), and Science and Health Joint Project of Chongqing (grant no. 2018QNXM015).

## Conflict of Interest

The authors declare that the research was conducted in the absence of any commercial or financial relationships that could be construed as a potential conflict of interest.
